# Ferrihydrite/ultrasound activated peroxymonosulfate for humic acid removal

**DOI:** 10.55730/1300-0527.3372

**Published:** 2022-02-23

**Authors:** Hang YANG, Yi ZHANG, Shibin XIA

**Affiliations:** 1 School of Resources and Environmental Engineering, Wuhan University of Technology, Wuhan, China; 2 State Key Laboratory of Freshwater Ecology and Biotechnology, Institute of Hydrobiology, Chinese Academy of Sciences, Wuhan, China

**Keywords:** Humic acid removal, ferrihydrite, ultrasound assistance, PMS activation, synergy index

## Abstract

In this study, ferrihydrite/ultrasound (US) system was used to activate peroxymonosulfate (PMS) to treat humic acid (HA) in artificial aqueous. The physical and chemical properties of ferrihydrite were characterized using SEM, zeta potential, BET, XRD, FTIR, and XPS analysis. A series of experiments were conducted to evaluate the effect of various factor on HA removal, including dosage of ferrihydrite, PMS concentration and pH value. The combination uses of US and ferrihydrite had obvious synergistic effect for HA removal. Under ferrihydrite/US/PMS system, nonthermal effect of US played the main role for HA removal. According to the result of radical quenching experiment, ^1^O_2_ was identified as the main reactive oxidative species (ROS) which contributed to HA removal. The study indicates ferrihydrite/US/PMS system is promising strategy for treatment of natural organic pollutant.

## 1. Introduction

Humic acid (HA) is a naturally occurring complex organic matter widely distributed in the natural environment. Excessive HA could cause serious problems in wastewater treatment plants due to the generation of carcinogenic disinfectant by-products like tri-halomethanes and haloacetic acids [1–3]. In addition, HA could be combined with organic pollutants and heavy metals, affecting the migration and conversion process of these pollutants [4,5]. Therefore, it is crucial to develop efficient and affordable technology to treat HA in artificial aqueous.

Advanced oxidation process (AOP) technology has been widely used in wastewater treatment, and related technologies have received increased attention [6–12]. A growing number of studies have focused on activating peroxymonosulfate (PMS) to generate SO_4_^•−^ and ^•^OH which can degrade pollutants [13–15]. The main strategies for PMS activation include alkaline, ultraviolet (UV), ultrasonic (US), microwave (MW), heat and transition metals catalysis [16–18]. Heterogeneous catalysts, especially iron-based materials have been widely employed to activate PMS due to the advantage of accessibility and low toxicity [11]. In addition, the combination uses of catalyst with other strategies for PMS activation to remove contaminant were worth studying due to the synergistic effect. US is a common auxiliary method to improve the activation efficiency of PMS. In our previous study, Fe_3_O_4_ particle was synthetized and employed for PMS activation to treat HA [19]. The result also indicated the combination uses of Fe_3_O_4_ and US had obvious synergistic effect for PMS activation.

Ferrihydrite is a promising iron-based mineral with the advantage of relatively large specific surface area and high stability. Compared to other iron-based materials, the cost of ferrihydrite was relatively low. At present, ferrihydrite has been widely employed in treatment of organic pollutants and heavy metals [20–24]. The application of ferrihydrite as catalyst for PMS activation is rarely studied [25].

Based on the above discussion, the combination uses of ferrihydrite and US to activate PMS for HA removal was proposed. The main aim of this work was to: (i) evaluate the synergistic effect of ferrihydrite and US for PMS activation to remove HA; (ii) identify the main reactive oxidative species (ROS) for HA removal; and (iii) understand the effects of thermal and nonthermal from US on HA removal.

## 2. Experimental

### 2.1 Materials and instruments

Materials: Ferrihydrite was bought from Runlong Environmental Materials Co., Ltd. HA was provided by the International Humic Acid Association. Potassium peroxymonosulfate (PMS), sulfuric acid, sodium hydroxide, ethanol, tert-butanol (TBA), dimethyl pyridine N-oxide (DMPO), 2,2,6,6-tetramethylpiperidine-1-oxyl (TEMPO), methyl phenyl sulfoxide (PMSO) and tryptophan were purchased from Sigma Aldrich. All chemicals were analytical grade and directly used without further purification. Ultrapure water (19.1 MΩ cm) was prepared using a Millipore water purification system.

Instruments: The crystal structure of ferrihydrite was analyzed using a particle X-ray diffraction (XRD, D8 Advance). X-ray photoelectron spectroscopy (XPS, EscaLab Xi+) measurement was employed to analyze the surface element value state of ferrihydrite. The morphology characterization of ferrihydrite was observed using a scanning electron microscope (SEM, JSM-IT300). Fourier transform infrared spectroscopy spectrum of ferrihydrite was recorded using a Fourier infrared spectrometer (FTIR, Nexus). A nanoparticle-sized zeta potential analyzer was used to analyze the zeta potential of ferrihydrite. The BET surface area and pore volume of ferrihydrite were determined by a Micromeritics ASAP 2020 system at (Mike 2020). A selfmade circulating water-cooling system was used to study the thermal effect during the operation of the ultrasound machine. Electronic paramagnetic resonance (EPR, Brook A300) was used to capture the produced ROS in EPR test, in which DMPO and TEMPO were used as a trapping agent. The concentration of iron leaching in the solution was measured by inductively coupled plasma mass spectrometer (ICP, perkinelmer). X-ray fluorescence spectrometer (PANalytical Axios) was employed to test the excitation emission matrix spectra (EEM) of HA solution. A UV-Vis spectrophotometer (UNICOWFUV-2) was used to test the concentration of the HA in solution. An ultrasound machine (KQ 100DE) was used to make ultrasonic radiation in experiments.

### 2.2 Experimental work

The batch experiments were conducted to explore the effect of experimental condition on HA removal. A total of 150 mL flask containing 100 mL of HA solution was employed throughout the experiment. The pH of the solution was adjusted using 1 mol/L H_2_SO_4_ and 1 mol/L NaOH. Under ferrihydrite/PMS system, the flask was set in a shaker with speed of 200 rpm at 25 °C. Under ferrihydrite/US/PMS system, the flask was set in an ultrasound machine with power of 100 W at 25 °C. At certain time intervals, 5 mL of the solution in the flask was collected and filtered with a syringe filter. The concentration of HA was analyzed using a UV spectrometer at 254 nm. In the cycle experiment of ferrihydrite, ferrihydrite was collected using centrifuge. All experiments were carried out for three times at least and the average value and error bar were employed.

### 2.3 Kinetic model, synergy index and removal rate of HA

The removal kinetics of HA was analyzed using the following pseudo-first-order kinetic model (Equation 1):


(1) $$\text{ln }({\text{C}}_{\text{t}}/{\text{C}}_{0})=-\text{kt}$$

where C_0_ and C_t_ are the initial HA concentration and residue concentration at time t, respectively; k represents the reaction rate constant (min^−^^1^).

The synergy index (SI) was used to justify the synergism or antagonism of two (or more) strategies for reaction process. When the value of SI > 1, the reaction process is synergism (positive synergistic effect); however, when the value of SI < 1, the reaction process is antagonism (negative synergistic effect). The synergy index of US and ferrihydrite for PMS activation to degrade HA was calculated according to Equation 2:


(2) $$\text{Synergy index}=\frac{{\text{k}}_{\text{US}/F\text{errihydrite}/pms}}{{\text{k}}_{\text{US}/\text{pms}}+{\text{k}}_{\text{Ferrihydrite}/pms}}$$

where k is the reaction rate constant (min^−^^1^) under respective system.

The removal rate of HA is obtained by calculation for Equation 3:


(3) $$\text{Removal rate}=\frac{{\text{C}}_{0}-{\text{C}}_{\text{t}}}{{\text{C}}_{0}}$$

where C_0_ and C_t_ are the initial HA concentration and residue concentration at time t, respectively.

### 2.4 Radical quenching experiment

Four radical quenching agents, including ethanol (EtOH), tert-butanol (TBA) and tryptophan, were used to quench the generated ROS in the experiment. Ethanol was used to quench SO_4_^•−^ and ^•^OH. TBA and tryptophan were used to quench ^•^OH and ^1^O_2_, respectively. The concentration of quenching agents was set as 400 mmol/L and 100 mmol/L in the experiment without and with US, respectively.

### 2.5 Thermal effect and nonthermal effect of US

Usually, the temperature in US tank increases with the irradiation time and the maximum temperature in the US tank was approximately 40 °C in this study. Therefore, to study the thermal effect form US radiation, the removal efficiency of HA using ferrihydrite/PMS at 40 °C was studied in shaker; to study the nonthermal effect form US radiation, a selfmade circulating water-cooling system was employed to maintain 25 °C in the US tank.

## 3. Characterization

### 3.1 Morphology and structure analysis of ferrihydrite

**Figure 1:**
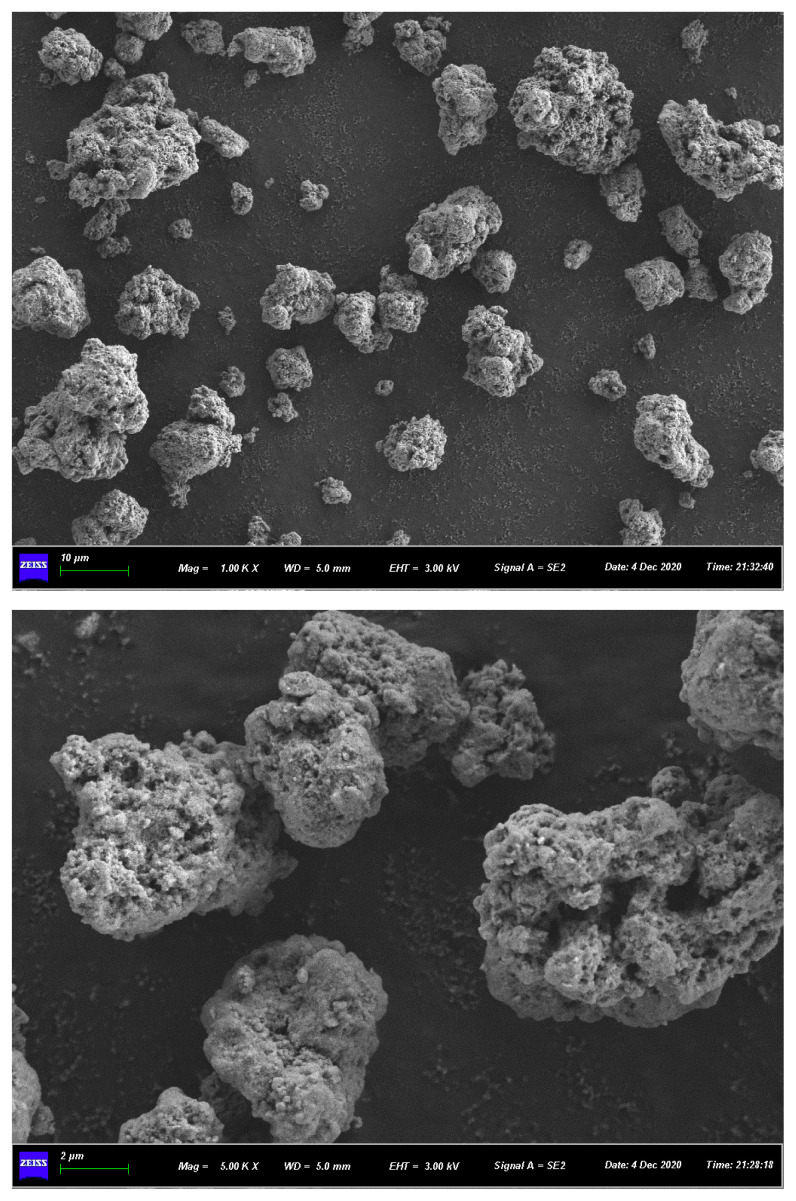
(a), (b) SEM image of ferrihydrite.

**Figure 2:**
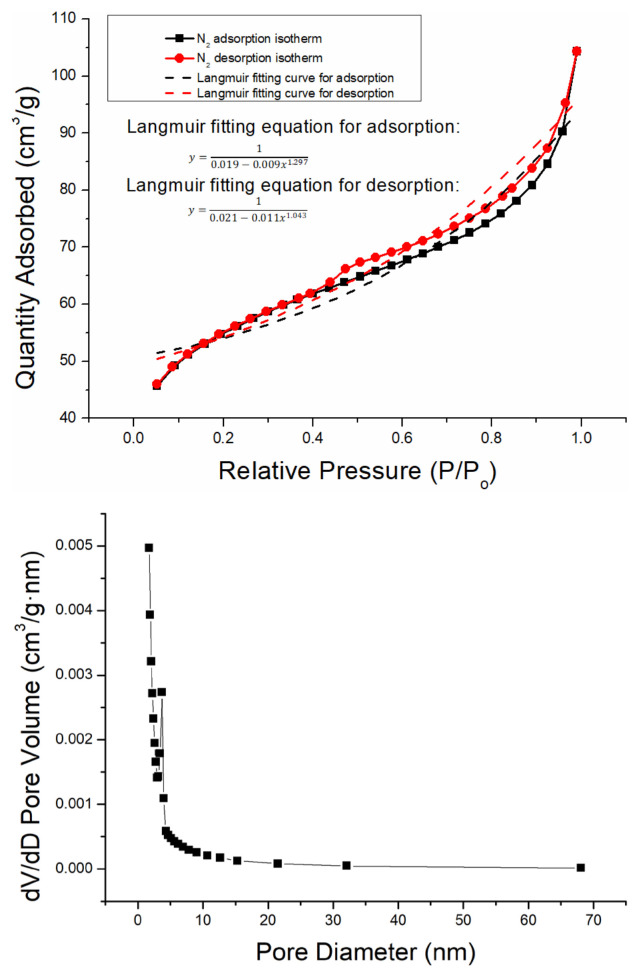
(a) Nitrogen adsorption-desorption isotherm and (b) pore distribution of ferrihydrite.

**Figure 3:**
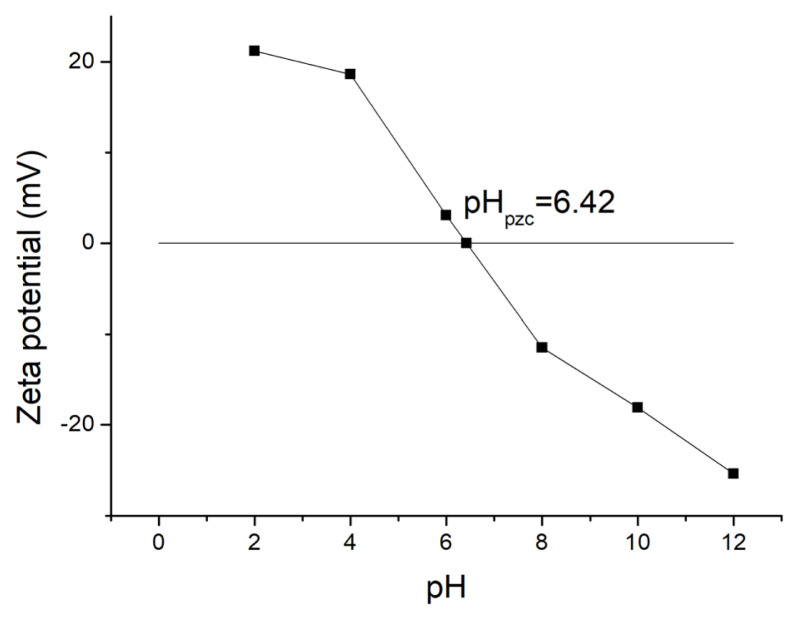
Zeta potential analysis for ferrihydrite.

Figures 1 shows the SEM image of ferrihydrite. The ferrihydrite exhibited irregular particle structure with rough surface and abundant pores. Figure 2 shows the N_2_ adsorption-desorption isotherm and pore distribution of ferrihydrite. Based on the IUPAC classification [26], the adsorption/desorption curve conformed to the II isotherm. The hysteresis loop of N_2_ adsorption isotherm belonged to the H3 type with no obvious adsorption saturation platform, which indicated the irregular pore structure of ferrihydrite. Table 1 shows the BET parameter of ferrihydrite. The specific area and average pore diameter of ferrihydrite was 179.39 m^2^/g and 4.4 nm, respectively. Figure 3 shows the zeta potential analysis of ferrihydrite. The zero point of charge (pH_pzc_) of ferrihydrite was calculated as 6.42, which indicated the surface of ferrihydrite was predominantly positive under pH < 6.42 while was predominantly negative under pH > 6.42.

### 3.2 Control experiment and kinetic analysis

**Figure 4:**
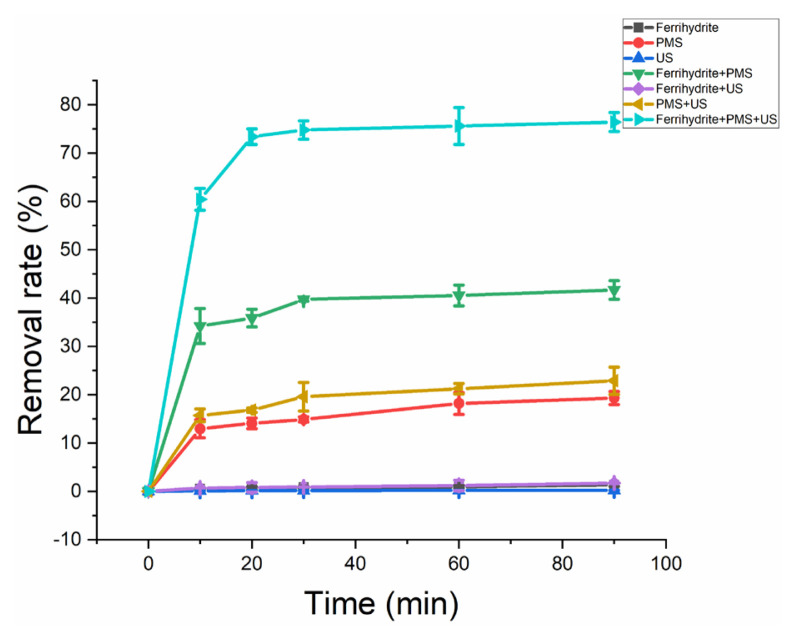
Removal rate of HA with different experimental modes. Experimental condition: dosage of ferrihydrite: 0.1 g/L; PMS concentration: 1 mmol/L; US power: 100 W; HA concentration: 10 mg/L; pH value: 7.

**Figure 5:**
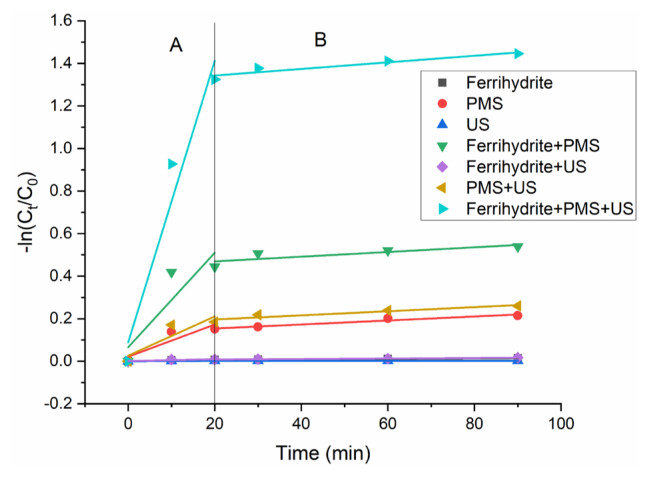
Removal kinetics of HA with different experimental modes. Experimental condition: dosage of ferrihydrite: 0.1 g/L; PMS concentration: 1 mmol/L; US power: 100 W; HA concentration: 10 mg/L; pH value: 7.

Figure 4 shows the removal efficiency of HA under different systems. The negligible HA removal was observed in the systems of only ferrihydrite, only US and ferrihydrite/US, which indicated the poor adsorption of HA on ferrihydrite. A certain of HA could be removal under PMS/US and only PMS systems, which indicated PMS could directly oxidize HA. Under PMS+ ferrihydrite reaction system, 41% of HA was removed after 90 min of reaction. However, in the US/PMS/ferrihydrite system, 76% of HA was removed after 90 min of reaction. HA was removed fast at first 20 min while the HA removal rate grown slowly after this step. Figure 5 and Table 2 show the kinetics plot and kinetic parameter of HA removal under different systems, respectively. According to Equation 2, synergy index of 2.9 was calculated from the combination uses of US and ferrihydrite, indicating the strong synergism of reaction process.

### 3.3 Effect of ferrihydrite dosage

**Figure 6:**
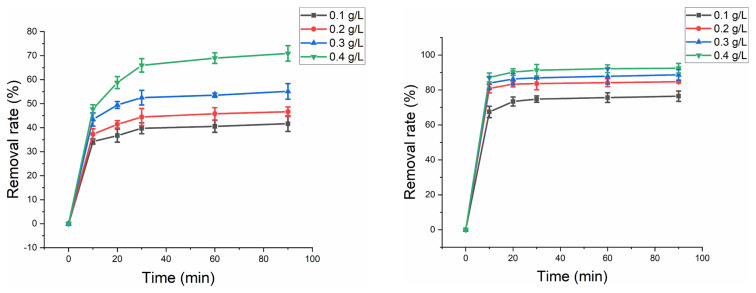
(a) Effect of different dosages of ferrihydrite on HA removal without and without US. Experimental parameters: dosage of ferrihydrite: 0.1–0.4 g/L; PMS concentration: 1 mmol/L; HA concentration: 10 mg/L; pH value: 7. (b) Effect of different dosages of ferrihydrite on HA removal with US. Experimental parameters: dosage of ferrihydrite: 0.1–0.4 g/L; PMS concentration: 1 mmol/L; US power: 100 W; HA concentration: 10 mg/L; pH value: 7.

Figure 6 shows the effect of ferrihydrite dosage on HA removal without and with US. For two systems, HA removal rate increased with the ferrihydrite dosage from 0.1 g/L to 0.4 g/L. With ferrihydrite dosage of 0.4 g/L, 70% and 91% HA of was removed without and with US, respectively. The increased ferrihydrite dosage could provide more active site to activate PMS, which resulted in higher HA removal. Considering removal efficiency and operation cost, 0.4 g/L dosage of ferrihydrite was selected for next experiment.

### 3.4 Effect of PMS concentration

**Figure 7:**
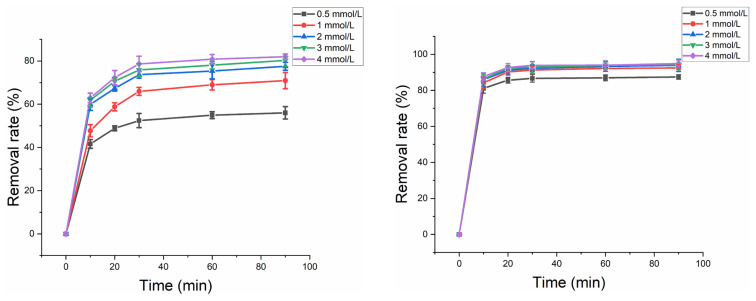
(a) Effect of different concentrations of PMS on HA removal without US. Experimental parameters: dosage of ferrihydrite: 0.4 g/L; PMS concentration: 0.5–4 mmol/L; HA concentration: 10 mg/L; pH value: 7. (b) Effect of different concentrations of PMS on HA removal with US. Experimental parameters: dosage of ferrihydrite: 0.4 g/L; PMS concentration: 0.5–4 mmol/L; US power: 100 W; HA concentration: 10 mg/L; pH value: 7.

Figure 7 shows the effect of PMS concentration on HA removal without and with US. For PMS/ferrihydrite system, the removal efficiency of HA enhanced with the increase of PMS concentration from 0.5 mmol/L to 4 mmol/L; for PMS/ferrihydrite/US system, the removal efficiency of HA enhanced and decreased subsequently with PMS concentration from 0.5 mmol/L to 4 mmol/L. Usually, higher PMS concentration could generate more ROS for HA removal. However, excessive PMS concentration could result in the selfscavenging effect, which quenched the generated ROS [27, 28]. The existence of US could amplify the selfscavenging effect, which impeded the HA removal with higher PMS concentration. Therefore, the optimal PMS concentration was 4 mmol/L and 1 mmol/L for HA removal without and with US, respectively.

### 3.5 Effect of initial pH

**Figure 8:**
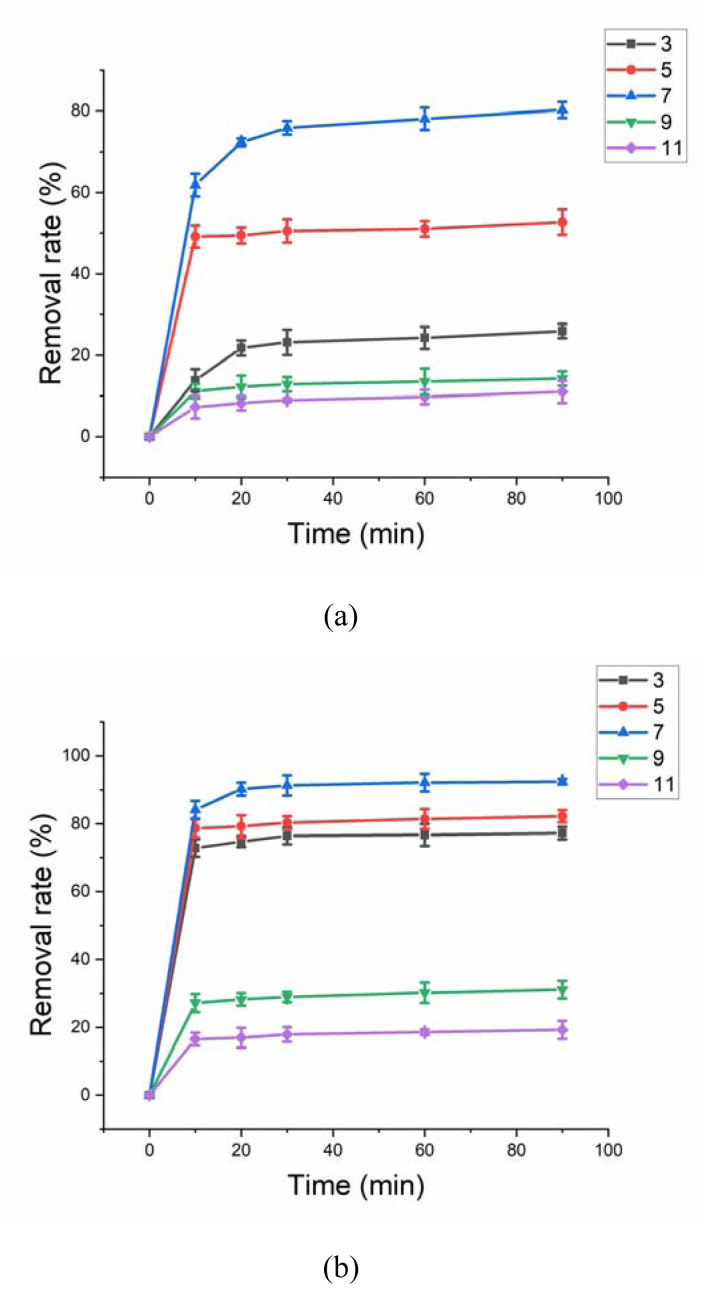
(a) Effect of different pH on HA removal without US. Experimental parameters: dosage of ferrihydrite: 0.4 g/L; PMS concentration: 4 mmol/L; HA concentration: 10 mg/L; pH value: 3–11. (b) Effect of different pH on HA removal with US. Experimental parameters: dosage of ferrihydrite: 0.4 g/L; PMS concentration: 1 mmol/L; US power: 100 W; HA concentration: 10 mg/L; pH value: 3–11.

Figure 8 shows the effect of pH on HA removal without and with US. For two systems, natural condition was the optimal pH for HA removal. PMS has two dissociation constants (0 and 9.4), the main formation of PMS in solution was HSO_5_^−^ and SO_5_^2^^−^under pH < 9.4 and pH > 9.4, respectively [29,30]. The pH_pzc_ of ferrihydrite was measured as 6.42. In the alkaline solution, the electrostatic repulsion existed between ferrihydrite and PMS, which was not conductive the activation of PMS, resulting the poor HA removal. In the acid solution, SO_4_^2−^ could be reacted with metal in ferrihydrite resulting in the reduction of activation site in ferrihydrite, which was also not conductive the activation of PMS. Therefore, the optimal pH for HA removal was neutral.

### 3.6 Thermal and nonthermal effects of US

**Figure 9:**
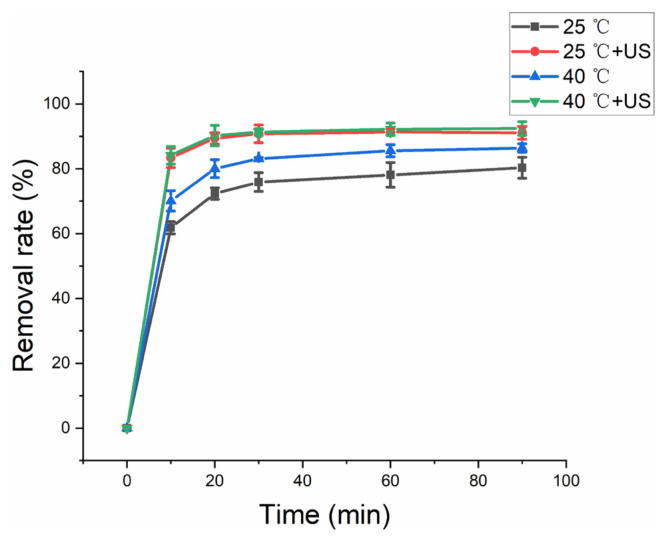
Effect of thermal effect and nonthermal effect on HA removal. Experimental parameters: dosage of ferrihydrite: 0.4 g/L; PMS concentration (without US): 4 mmol/L; PMS concentration (with US): 1 mmol/L; US power: 100 W; HA concentration: 10 mg/L; pH value: 7.

Thermal and nonthermal effects are important feature of US, because the temperature in US tank usually increases with the process of reaction [31–33]. In the PMS activation system, heat is an effective method to activate PMS. However, excessive temperature results in more cavitation collapse in US system, which reduces the US energy and synergy activation for PMS. The thermal effect and nonthermal effect for HA removal were investigated (Figure 9). For PMS/ferrihydrite system, enhanced temperature resulted in the high HA removal rate due to the heat activation of PMS. However, for PMS/ferrihydrite/US system, the HA removal rate had no obvious change, which indicated nonthermal effect played an important role for HA removal.

### 3.7 Recycling capacity and stability of ferrihydrite

**Figure 10:**
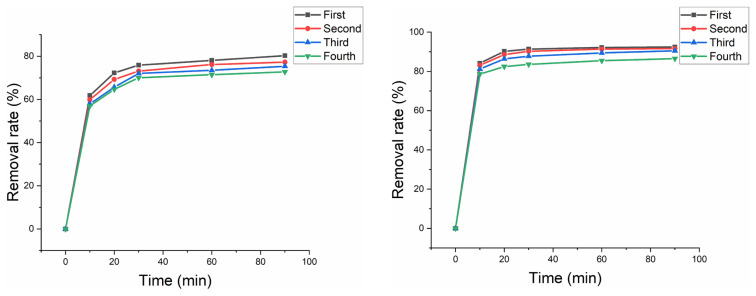
Cycle utilization of ferrihydrite: (a) cycle utilization of ferrihydrite without US and (b) cycle utilization of ferrihydrite with US.

**Figure 11:**
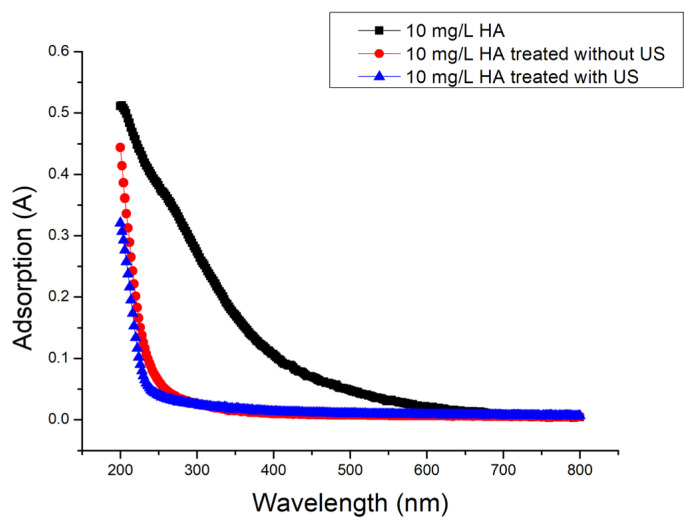
UV-Vis of 10 mg/L HA solution: (a) untreated HA solution, (b) treated HA solution by ferrihydrite/PMS without US, and (c) treated HA solution by ferrihydrite/PMS with US.

**Figure 12:**
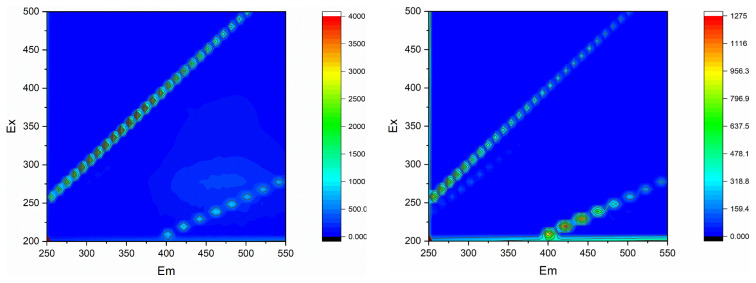
EEM of 10 mg/L HA solution.

The cycle capacity of ferrihydrite for ferrihydrite/PMS and ferrihydrite/PMS/US was examined using cycle experiments (Figure 10). The removal rate of HA slight decreased after four cycles, which was due the weight loss during the centrifugal recovery process. According to the result of control experiment (Figure 4), ferrihydrite had shown poor adsorption for HA. Therefore, adsorption/desorption processes of HA would rarely be involved in the repeated use of ferrihydrite. ICP-OES was used to analyze Fe ion leaching concentration of ferrihydrite. Under condition of without and with US, the Fe ion leaching concentration of ferrihydrite was 0.177 mg/L and 0.118 mg/L, respectively. UV-Vis and EEM were used to analyze HA solution (Figures 11 and 12), in which the characterization region of HA was greatly reduced, indicating the considerable HA removal was obtained.

**Figure 13:**
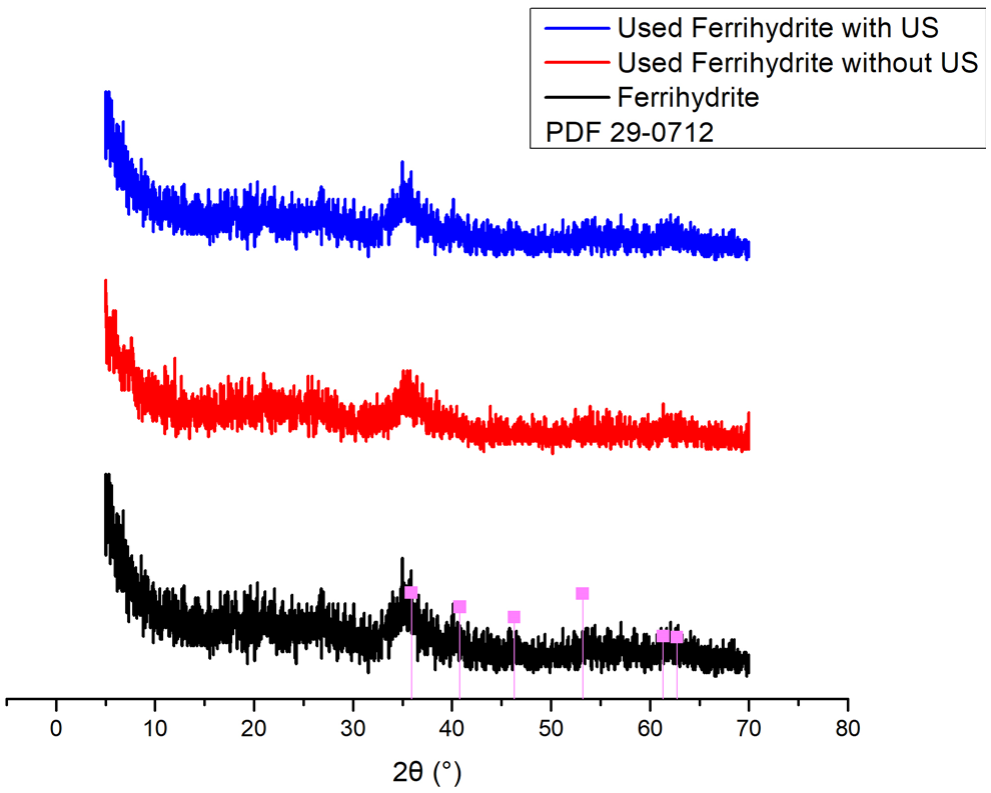
XRD pattern of ferrihydrite.

Figure 13 shows XRD pattern of ferrihydrite. The main characterization peak for ferrihydrite was observed at 2θ = 35° (indexed to PDF 29-0712). The other characterization peak was not detected, which was ascribed to the poor crystal structure of ferrihydrite and the presence of impurity in ferrihydrite. The XRD patterns of used ferrihydrite was consistent with that of original ferrihydrite, which indicated the stable structure of ferrihydrite.

**Figure 14:**
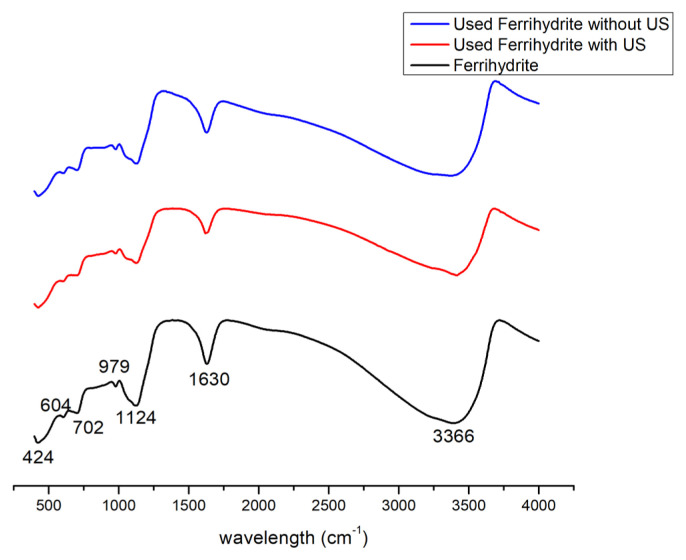
FTIR spectrum of ferrihydrite.

Figure 14 shows the FTIR spectra of ferrihydrite ranged from 4000 cm^−^^1^ to 400 cm^−^^1^. The broad band at 3366 cm^−^^1^ was ascribed to O-H stretching vibration of hydroxyl groups of ferrihydrite [34]. The strong peak appeared at 1630 cm^−^^1^ was assigned to O-H bending vibration in ferrihydrite [35]. The weak peak at 604 cm^−^^1^ was corresponding to Fe–O transverse vibration peak [36]. No obvious change was observed in the FTIR spectra of used ferrihydrite.

**Figure 15:**
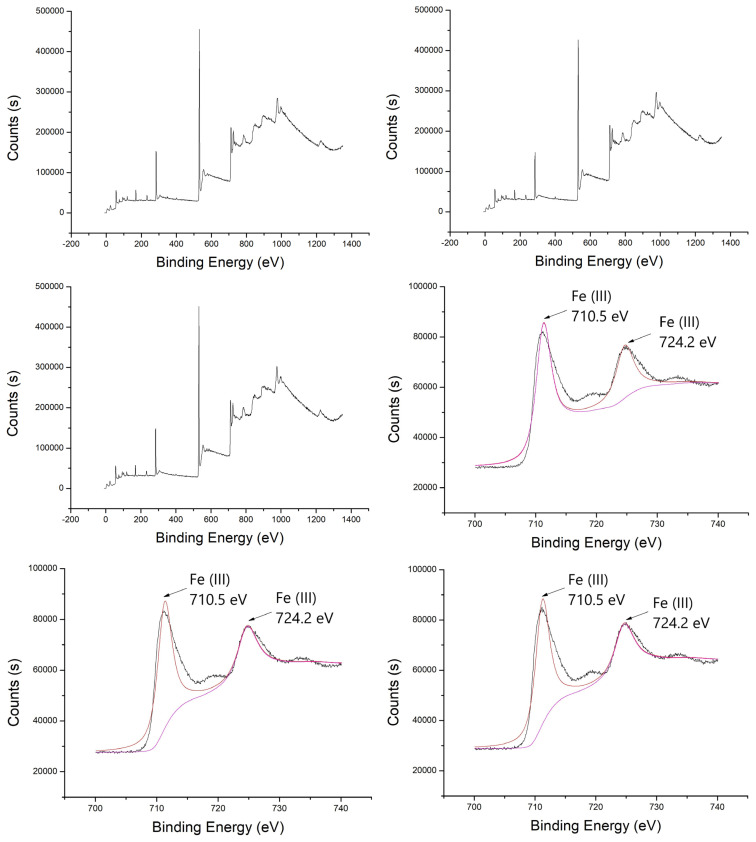

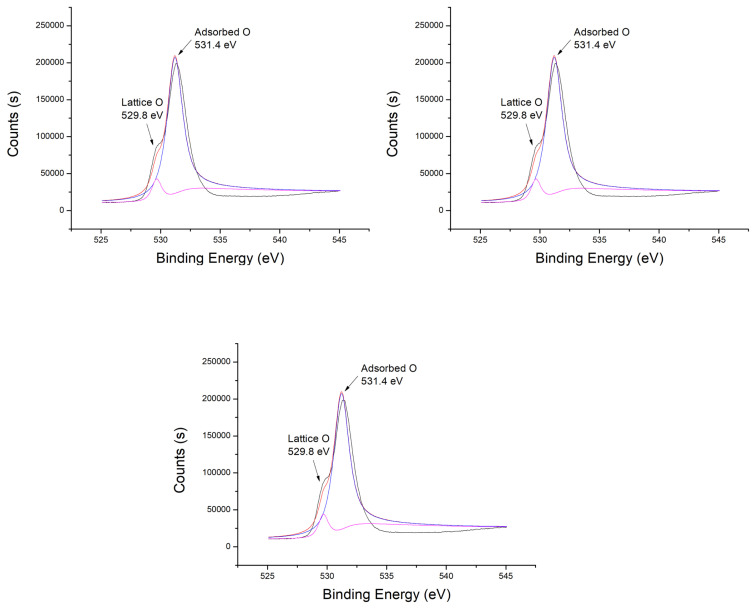
XPS analysis of ferrihydrite with (a) survey of original ferrihydrite, (b) survey of used ferrihydrite without US, (c) survey of used ferrihydrite with US, (d) Fe_2p_ of original ferrihydrite, (e) Fe_2p_ of used ferrihydrite without US, (f) Fe_2p_ of used ferrihydrite with US, (g) O 1s of original ferrihydrite, (h) O 1s of used ferrihydrite without US, and (i) O 1s of used ferrihydrite with US.

Figure 15 shows the XPS spectra of ferrihydrite. The surface of ferrihydrite was mainly consisted of Fe and O element. The binding energies at 724.2 eV and 710.5 was assigned to Fe _2p1/2_ and Fe _2p3/2_, respectively, which were corresponding to the Fe^3+^[37]. The binding energies at 529.8 eV and 531.4 eV in the O 1s pattern was ascribed to adsorbed oxygen in Fe-O-Fe and lattice oxygen in Fe-O–H, respectively. After used for reaction, the peak intensity and place were still unchanged, which indicated the excellent stability of ferrihydrite.

The results of XPS, XRD and FTIR analysis of ferrihydrite demonstrated the structure composition, surface group and surface element valence had no obvious change after use. The result indicated the good recycling and stability of ferrihydrite.

### 3.8 Radical quenching test and ESR test

**Figure 16:**
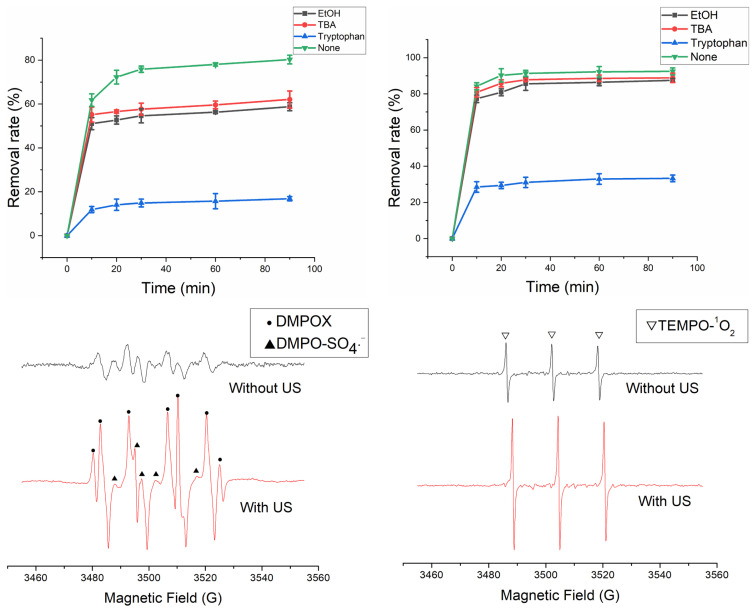
Radical quenching tests (a) without US and (b) with US; ESR tests for (c) SO_4_^•−^, ^•^OH, and (d) ^1^O_2_.

In order to determine the contribution of the ROS responsible for the degradation of HA. The effects of SO_4_^•−^, ^•^OH and ^1^O_2_ for HA removal were studied using different quenchers [38–40]. As shown in Figures 16a and 16b, SO_4_^•−^, ^•^OH and ^1^O_2_ directly contributed to HA removal and ^1^O_2_ was the main contributor. Equations 4–9 indicated the possible production pathway for ROS.

Figures 16c and 16d show the EPR test with and without US. The ERP signal intensity in ferrihydrite/PMS/US system was higher than that in ferrihydrite/PMS system. The signals of DMPOX, DMPO-SO_4_^•−^ and TEMPO-^1^O_2_ were observed, in which DMPOX was formed by DMPO trapping two hydroxyl groups[15]. The result of EPR test indicated the generation of SO_4_^•−^, ^•^OH, and ^1^O_2_ in the ferrihydrite/PMS system and ferrihydrite/PMS/US system. In addition, the generated quantity of SO_4_^•−^, ^•^OH, and ^1^O_2_ was higher in ferrihydrite/PMS/US system than that in the ferrihydrite/PMS system.


(4) $${\text{HSO}}_{5}^{-}\to {\text{H}}^{+}+{\text{SO}}_{5}^{2-}$$

(5) $${\text{SO}}_{5}^{-}+{\text{H}}_{2}\text{O}\to {\text{O}}_{2}^{•-}+{\text{SO}}_{4}^{2-}+{\text{H}}^{+}$$

(6) $${\text{O}}_{2}^{•-}+2{\text{H}}_{2}\text{O}\to 10_{2}+{\text{H}}_{2}{\text{O}}_{2}+2{\text{OH}}^{-}$$

(7) O•H+H2O→HO2+H2O

(8) $${\text{HO}}_{2}\to {\text{H}}^{+}+{\text{O}}_{2}^{•-}$$

(9) O2•-+O•H→102+OH-

## 4. Conclusion

Ferrihydrite were employed to activate PMS for HA removal. The use of US had strong synergetic effect for HA removal with a synergy index of 2.9. The result of control experiment indicated higher HA removal efficiency was achieved with higher dosage of ferrihydrite and appropriate PMS concentration under neutral condition. The thermal and nonthermal effects from US both resulted in HA removal and nonthermal effect had played the most important role. EEM and UV-Vis data illustrated the obvious HA removal in ferrihydrite/PMS/US system. SO_4_^•−^, ^•^OH and ^1^O_2_ were responsible to the removal of HA and and ^1^O_2_ was the dominant ROS for HA removal. This study indicates ferrihydrite/US/PMS was an effective method for HA removal.

## Figures and Tables

**Table 1:** BET parameters for ferrihydrite.

Sample	Specific area (m^2^/g)	Volume (cm^3^/g)	Average pore diameter (nm)
Ferrihydrite	179.39	0.16	4.4

**Table 2:** The kinetic parameter with different experimental modes.

Constant	Only ferrihydrite	Only PMS	Only US	Ferrihydrite+ PMS	Ferrihydrite+ US	PMS+ US	Ferrihydrite+ PMS + US
K_a_ (min^−^^1^)	3.66943E-4	0.00758	8.13211E-5	0.02221	4.22939E-4	0.00922	0.06621
K_b_ (min^−^^1^)	9.4486E-5	9.3943E-4	9.1976E-6	0.0011	1.24878E-4	9.67464E-4	0.00155

## Data Availability

All data, models, and code generated or used during the study appear in the submitted article.
